# Potential of UAV-Based Active Sensing for Monitoring Rice Leaf Nitrogen Status

**DOI:** 10.3389/fpls.2018.01834

**Published:** 2018-12-14

**Authors:** Songyang Li, Xingzhong Ding, Qianliang Kuang, Syed Tahir Ata-UI-Karim, Tao Cheng, Xiaojun Liu, Yongchao Tian, Yan Zhu, Weixing Cao, Qiang Cao

**Affiliations:** ^1^National Engineering and Technology Center for Information Agriculture (NETCIA), Key Laboratory for Crop System Analysis and Decision Making, Ministry of Agriculture, Jiangsu Key Laboratory for Information Agriculture, Jiangsu Collaborative Innovation Center for the Technology and Application of Internet of Things, Nanjing Agricultural University, Nanjing, China; ^2^Key Laboratory of Soil Environment and Pollution Remediation, Institute of Soil Science, Chinese Academy of Sciences, Nanjing, China

**Keywords:** active canopy sensor, RapidSCAN, red edge, ultra low-level airborne, sensing distance evaluation

## Abstract

Unmanned aerial vehicle (UAV) based active canopy sensors can serve as a promising sensing solution for the estimation of crop nitrogen (N) status with great applicability and flexibility. This study was endeavored to determine the feasibility of UAV-based active sensing to monitor the leaf N status of rice (*Oryza sativa* L.) and to examine the transferability of handheld-based predictive models to UAV-based active sensing. In this 3-year multi-locational study, varied N-rates (0–405 kg N ha^−1^) field experiments were conducted using five rice varieties. Plant samples and sensing data were collected at critical growth stages for growth analysis and monitoring. The portable active canopy sensor RapidSCAN CS-45 with red, red edge, and near infrared wavebands was used in handheld mode and aerial mode on a gimbal under a multi-rotor UAV. The results showed the great potential of UAV-based active sensing for monitoring rice leaf N status. The vegetation index-based regression models were built and evaluated based on Akaike information criterion and independent validation to predict rice leaf dry matter, leaf area index, and leaf N accumulation. Vegetation indices composed of near-infrared and red edge bands (NDRE or RERVI) acquired at a 1.5 m aviation height had a good performance for the practical application. Future studies are needed on the proper operation mode and means for precision N management with this system.

## Introduction

Nitrogen (N) plays a vital role in improving crop growth and productivity (Novoa and Loomis, 1981; Ata-Ul-Karim et al., 2016). Over 200 million tons of N fertilizers are estimated to be used in 2018 and continue to increase at 1.8% per year (FAO (Food and Agriculture Organization of the United Nations), 2015). However, over-application of N fertilizers is the alarming issue that has caused low N use efficiency, leading to N deposition and water eutrophication (Conant et al., 2013; Liu et al., 2013; Huang et al., 2017). Therefore, it is imperative to develop highly efficient, reliable and practical methods for monitoring crop N status to meet the demand for precision N management (Miao et al., 2011). Several traditional methods, such as the leaf color chart (Alam et al., 2005) or destructive chemical analysis (Asner and Martin, 2008) are limited by low efficiency, small-scale applicability, and professional experience requirements for accurate diagnosis. With the advances of optical sensors and remote sensing technology, crop N-status monitoring, and management based on the spectrum has been widely used in different crops (Saberioon et al., 2014; Padilla et al., 2018).

Many studies have been conducted on the utilization of spaceborne and airborne passive remote sensing for crop monitoring. Satellite remote sensing provides great possibility for large-scale crop growth monitoring and precision management, for example, satellites images of FORMOSAT-2 were used for rice (*Oryza sativa* L.) N-status monitoring (Huang et al., 2015); however, the quality of remote sensing images from passive sensor-based satellites would be affected by bad weather conditions like cloud and fog, leading to the lack of applicable in-season sensing datasets for crop monitoring. The unmanned aerial vehicle (UAV) emerges as a promising remote sensing platform owing to its flexibility (Yang et al., 2017), and it was widely investigated for crop monitoring with imaging sensors (Maresma et al., 2016). Rice grain yield and leaf area index (LAI) were predicted by multi-temporal vegetation indices (VIs) from UAV-based multispectral imagery (Zhou et al., 2017), and the red edge (720 nm) and near-infrared (800 nm) band-based VIs were found to be more effective in the prediction of yield and LAI. However, despite the development of semi-automatic procedures, image processing, and analysis have still been too specialized and challenging for ordinary consumers until now.

Non-imaging optical canopy sensors directly collect standardized spectral reflectance with great flexibility in data achievement and processing over imaging sensors. Sensitive wave bands and VIs have been previously utilized for crop N-status estimation using passive hyperspectral canopy sensors (Tian et al., 2014). In addition, passive multispectral sensors were also developed for crop monitoring (Ni et al., 2016), and some of them were mounted on ground vehicle platforms (Pei et al., 2014). Ni et al. (2017) designed a UAV-mounted crop-growth monitoring system based on a passive sensor with a red band and a near-infrared band, and it was proved to have potential for predicting wheat leaf nitrogen status with Normalized Difference Vegetation Index (NDVI) and Ratio Vegetation Index (RVI).

Active sensors were developed with an internal light source to avoid the calibration requirements for illumination and the light angle (Holland et al., 2012). One of the superiorities is their potential to solve the problems of cloud cover and time limitations for measurements, which limit the use of passive sensors under such conditions (Stamatiadis et al., 2010). Most studies focus on traditional two-band active canopy sensors (Danielw and Johne, 2010; Samborski et al., 2016), but three-band active canopy sensors like Crop Circle ACS-470 (Holland Scientific Inc., Lincoln, NE, USA) were reported to improve the estimating performance of winter wheat or rice N status as compared to two-band sensors (Cao et al., 2015; Shi et al., 2015). RapidSCAN CS-45 (Holland Scientific Inc., Lincoln, NE, USA) is a small-sized portable three-band active sensor which has been used in precision agriculture. The previous study on rice indicated that VIs calculated from RapidSCAN wavebands could diagnose the rice N nutrition index well (Lu et al., 2017). In addition, studies on wheat, maize, soybean, and potato and soybean also showed the potential of the handheld RapidSCAN sensor to monitor crop N-status, to predict grain yield, and for cultivar selection, as well as for making nitrogen fertilizing recommendation (Bonfil, 2016; Aranguren et al., 2018; Miller et al., 2018).

Consequently, UAV-based active sensing is expected to offer flexibility, affordability, and applicability for large-scale monitoring compared to handheld active sensing. In addition, researchers paid much attention to combining different sensing data for establishing universal sensing approaches which are suitable for a better sensing performance or wider scale application (Gevaert et al., 2015; Schirrmann et al., 2017). With a unique character of using the same kind of sensor with a similar sensing height, UAV-based active sensing has implied a hypothesis of transferring handheld-based predictive models to UAV-based active sensing.

However, considering previous studies with manned aircraft (Lamb et al., 2009, 2014) and unmanned aerial vehicles (Krienke et al., 2017), still, little attention has been paid to investigate the possibility of applying active sensors on ultra low-altitude aerial vehicles. A potential issue for these studies was the effective sensing distance to accurately collect crop canopy reflectance. A unique feature of the RapidSCAN CS-45 is its ability to conduct height-independent spectral reflectance measurements named Pseudo Solar Reflectance (PSR) measurements introduced by its manufacturer. Based on this sensor specialty, Krienke et al. (2017) utilized the RapidSCAN CS-45 on a UAV platform for testing its applicable mode and proving its performance when evaluating maize N variability. In spite of the technical potential, sensing distance evaluation for practical application is still necessary.

Although the active canopy sensor RapidSCAN CS-45 has been proved to have applicability for diagnosing rice N-status using handheld mode (Lu et al., 2017), the potential of UAV-based active sensing for rice N-status monitoring has not yet been tested. Therefore, the objectives of the current study were two-fold: (1) to determine whether UAV-based active sensing is feasible to monitor rice leaf N-status and (2) to examine the transferability of handheld-based predictive models to UAV-based active sensing.

## Materials and Methods

### Study Area and Experimental Design

**Figure 1:**
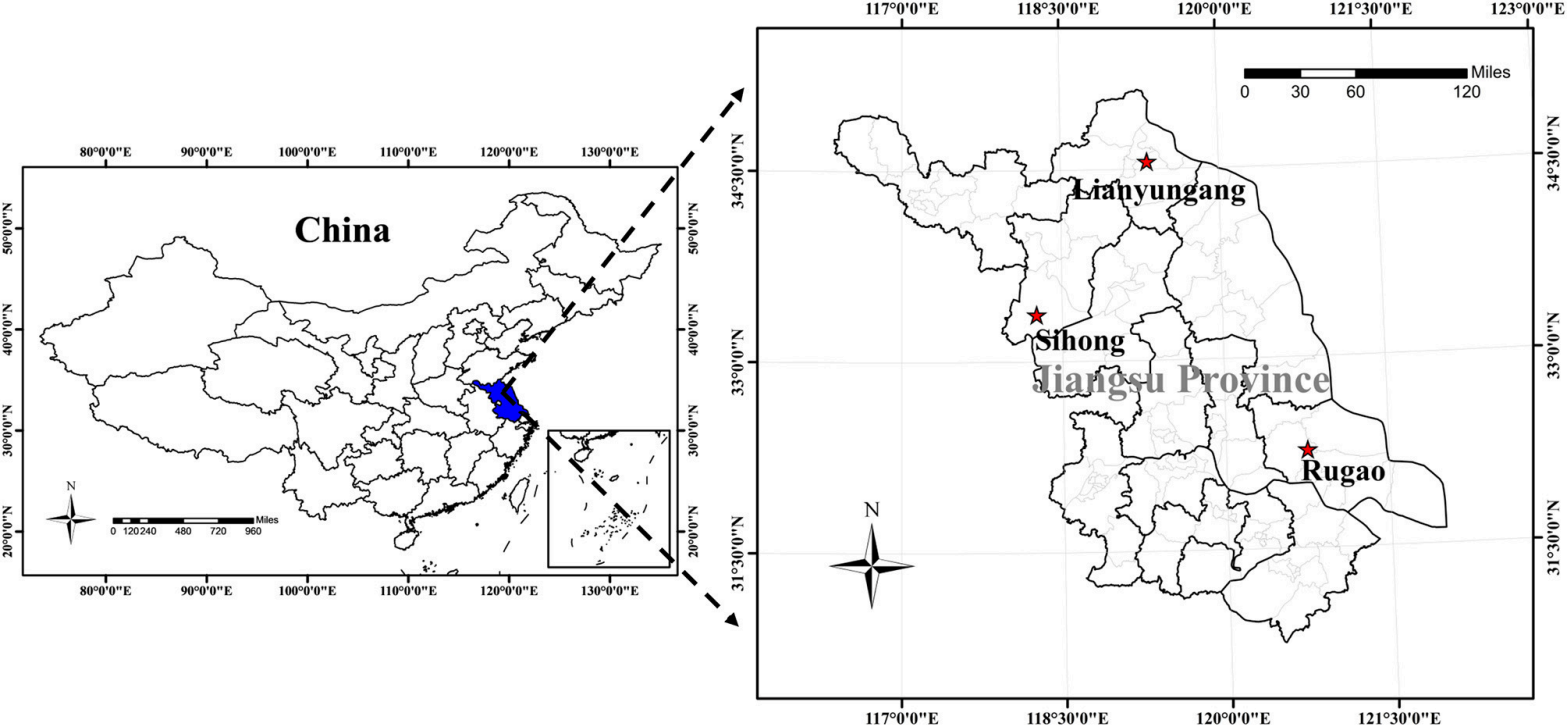
Study site: rice experiments conducted at Sihong, Lianyungang, and Rugao Experimental Station in Jiangsu Province of China.

Field trials were carried out over three rice growing seasons (June–October 2015–2017) in Jiangsu Province of east China, which is a traditional rice-farming area with a long rice planting history (Figure 1). Trials were established at Rugao Experimental Station (32.27°N and 120.75°E, central-eastern Jiangsu) in 2015 and 2016, Sihong Experimental Station (33.37°N and 118.26°E, northern Jiangsu) in 2016, and Lianyungang Experimental Station (34.56°N and 119.32°E, northern Jiangsu) in 2017. Detailed information is presented in Table 1.

**Table 1:** Description of field experiments conducted for calibration and validation.

**Experiment**	**Sensing mode**	**Location**	**Varieties**	**Number of samples**	**Transplanting date**	**Growth stages of sensing and sampling with DAT (d)**
**CALIBRATION EXPERIMENTS**						
Experiment 1 2016	Hand-held	Sihong	Lianjing-7, Wuyunjing-24, Ningjing-4	288	25 June	TI (27, 33), SE (39, 47), BT (54, 60), HD (76), FI (96)
Experiment 2 2017	Hand-held	Lianyun gang	Lianjing-15, Zhongdao-1	336	19 June	TI (27), SE (38, 45), BT (52, 62), HD (80), FI (90)
**VALIDATION EXPERIMENTS**						
**Handheld Sensing**						
Experiment 3 2015	Hand-held	Rugao	Wuyunjing-24	72	15 June	TI (29), SE (39, 45), BT (52, 57, 62)
Experiment 4 2016	Hand-held	Rugao	Wuyunjing-24, Ningjing-4	96	15 June	SE (40), SE (48), BT (57), HD (67)
**UAV-Based Sensing**						
Experiment 2 2017	UAV-based	Lianyun gang	Lianjing-15, Zhongdao-1	192	19 June	SE (45), BT (52, 62),HD (80)

*DAT represents days after transplanting of each sensing and sampling procedure. TI, SE, BT, HD, and FI represent the growth stage of tillering, stem elongation, booting, heading, and filling, respectively*.

Experiment 1 was conducted at Sihong Experimental Station in 2016, which covered four N rates (0, 120, 240, 360 kg N ha^−1^) and three rice varieties. Experiment 2 was conducted at Lianyungang Experimental Station in 2017, which covered four N rates (0, 135, 270, 405 kg N ha^−1^), two transplanting ways (pot-seedling and carpet-seedling mechanical transplanting), and three rice varieties. Experiment 3 was conducted at Rugao Experimental Station in 2015, which covered four N rates (0, 60, 150, 240 kg N ha^−1^) and one rice variety. Experiment 4 was conducted at Rugao Experimental Station in 2016, which covered four N rates (0, 100, 250, 400 kg N ha^−1^) and two rice varieties.

All field experiments were arranged in a randomized complete block design with three replicates. Each plot size was 56 m^2^ (7 × 8 m) in Experiment 1, 120 m^2^ (8 × 15 m) in Experiment 2, and 35 m^2^ (5 × 7 m) in Experiment 3 and Experiment 4. N fertilizer in all field experiments was applied in the form of granular urea as three splits: 50% before transplanting, 30% at the tillering stage, and 20% at the booting stage. For each plot, based on soil analysis and recommendations from the local agriculture department, 127 kg P_2_O_5_ ha^−1^ was applied before transplanting in the form of Ca(H_2_PO_4_)_2_ and 225 kg K_2_O ha^−1^ was applied as two splits: 50% before transplanting and 50% at the stem elongation stage. Carpet rice seedlings (for all the experiments) and pot rice seedlings (for Experiment 2) were prepared in seedling fields and transplanted into the experimental fields.

### Active Canopy Sensor Data Collection

The active optical crop canopy sensor RapidSCAN CS-45 with three wavebands, including red (R, 670 nm), red-edge (Re, 730 nm), and near-infrared (NIR, over 780 nm) regions, was used in this study. Spectral reflectance (%) of each band and GPS data can be automatically collected and recorded in the memory module of the sensor at 2.5 Hz (one reading per 0.4 s) by modifying the logging method of the sensor. Data can be exported as a .csv file by PC software. Owing to the lightweight (0.8 kg) and wide measurement range (0.3 to 3 m height above the rice canopy) of the sensor, it is theoretically feasible to mount it on the UAV for practical application.

**Figure 2:**
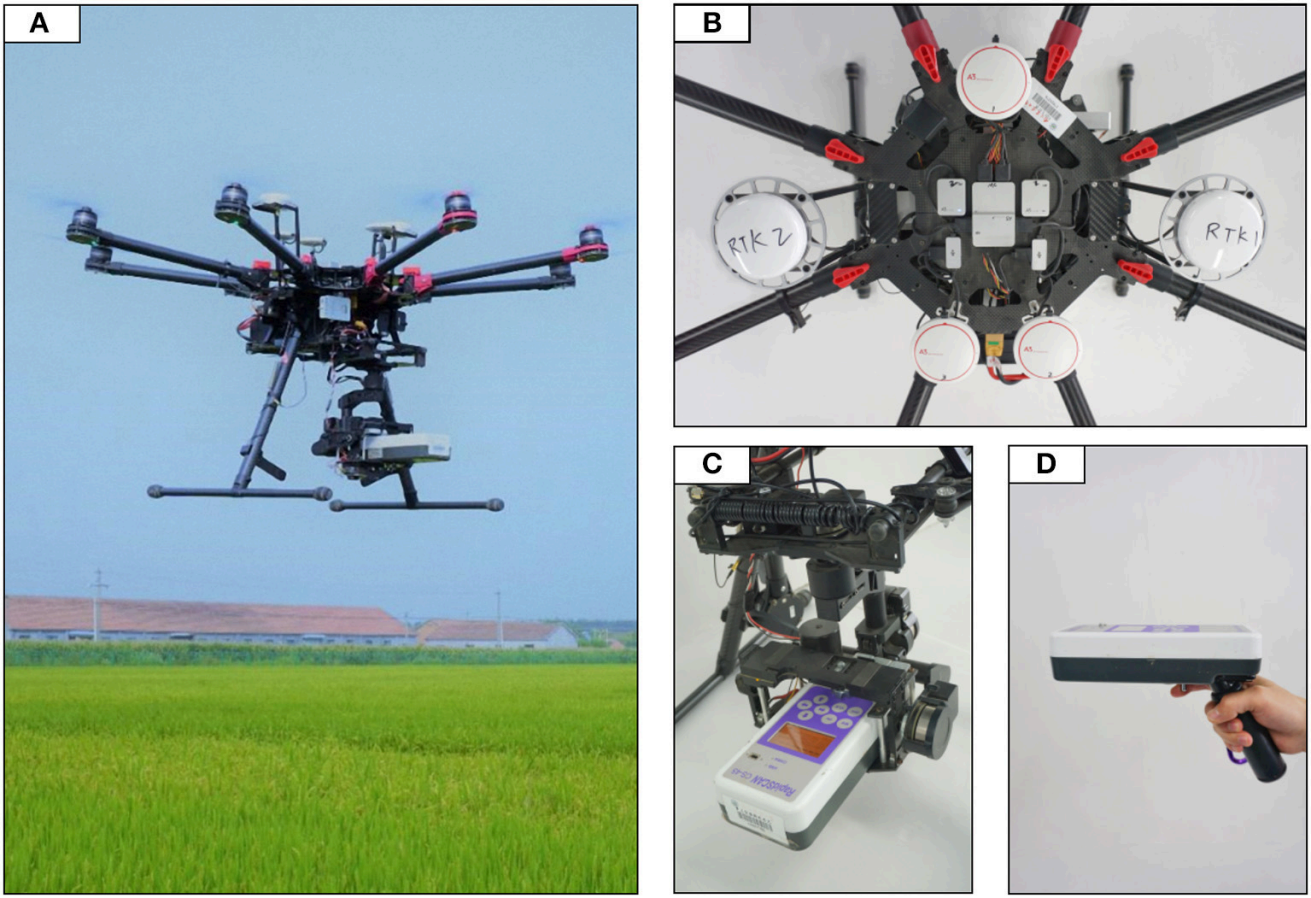
Overview of the sensing equipment used in this study. **(A)** Spreading Wings S1000+ used as the sensing platform for low-altitude rice monitoring; **(B)** Flight controller module with DJI D-RTK GNSS system; **(C)** RapidSCAN CS-45 sensor mounted on a customized gimbal under the UAV; **(D)** RapidSCAN CS-45 sensor in handheld mode for data acquirement.

The multi-rotor UAV Spreading Wings S1000+ (DJI-Innovations Inc., Shenzhen, China) with DJI D-RTK GNSS system was used to provide a stable flight condition with accurate centimeter-level 3D positioning (Figure 2). The RapidSCAN CS-45 sensor was mounted on a customized gimbal in a fixed sensing posture under the UAV. In Experiment 2, controlled by the ground station program, the UAV aviated automatically according to the pre-concerted flight path along the central axis in the row direction of each plot with heights of 1.5 and 2 m above the canopy (resulting in 0.33 and 0.58 m^2^ view area), respectively. The heading speed of the UAV was set as 2 m/s.

Handheld sensing in all experiments was conducted by the operator on approximately the same path as that of the UAV-based sensing using RapidSCAN CS-45. Sensor readings were collected ~1–1.5 m above the rice canopy (resulting in 0.15–0.33 m^2^ view area).

Sensor data were processed in ArcMap 10.5 (ESRI, Redlands, CA, USA) and assigned to different sampling plots with the GPS position of each reading point. A buffer of 1 m was utilized to exclude data near the plot boundary. The average reflectance values collected by RapidSCAN CS-45 were computed to represent each plot both in UAV and handheld-based sensing. Calculated spectral vegetation indices used in this study are listed in Table 2.

**Table 2:** Summary of the calculated spectral vegetation indices (VI) selected for this study.

**VI**	**Formula**	**References**
Normalized difference red edge (NDRE)	(NIR–Re)/(NIR+Re)	Barnes et al., 2000
Red edge ratio vegetation index (RERVI)	NIR/Re	Jasper et al., 2009
Normalized difference vegetation index (NDVI)	(NIR–R)/(NIR+R)	Rouse et al., 1973
Ratio vegetation index (RVI)	NIR/R	Jordan, 1969

*R, Re, and NIR indicate reflectance (%) collected by RapidSCAN at the band region of red, red edge, and near infrared, respectively*.

### Plant Sampling and Measurement

Rice plant samples were acquired right after collecting sensing data. Destructive plant samples of above-ground parts were randomly collected (three hills per plot) from the sensed plants according to the average number of tillers. The samples were separated into leaves and stems. The separated leaves were scanned by Li-3000c (Li-Cor., Lincoln, NE, USA) to determine the leaf area index (LAI). Each sampled component was put into the oven for enzyme deactivation under 105°C for half an hour, and then under 80°C for 72 h for weighing. The weight of each leaf sample was used to determine the leaf dry matter (LDM). Leaf nitrogen concentration (LNC) was determined by the micro-Kjeldahl method (Bremner and Mulvaney, 1982). Leaf nitrogen accumulation (LNA) was calculated by multiplying LDM and LNC. LDM, LAI, and LNA were selected as the nitrogen indicators in this study.

### Data Analysis

As shown in Table 1, to test the possibility of building universal predictive models for different applications, the handheld datasets from Experiment 1 and Experiment 2 were used for VI-based model calibration. The handheld datasets from Experiment 3 and Experiment 4 were used to validate the regression models. For examining the transferability of the models from handheld to UAV sensors, the models derived from the handheld data were applied to the UAV data from Experiment 2. Besides, the validation on UAV data were performed for two sensing heights (1.5 and 2 m above the canopy) to evaluate the stability of the UAV system over two sensing distances.

The mean value, standard deviation (SD), and coefficient of variation (CV, %) of rice agronomic parameters were calculated using SPSS 25 (SPSS Inc., Chicago, IL, USA). For simplicity and VI comparison purpose, N indicators were first predicted with single VI-based models. Therefore, linear and three types of non-linear models (quadratic, exponential, and power) were built and evaluated between each VI and each N indicator (LDM, LAI, and LNA). The basic function forms of linear (Equation 1), quadratic (Equation 2), exponential (Equation 3), and power (Equation 4) regressions are given by:

(1) $$\begin{array}{r} y=a_{0}\;+\;a_{1}x \end{array}$$

(2) $$\begin{array}{r} y=b_{0}\;+\;b_{1}x+\;b_{2}x^{2} \end{array}$$

(3) $$\begin{array}{r} y=c_{0}e^{c_{1}x} \end{array}$$

(4) $$\begin{array}{r} y=d_{0}\;x^{d_{1}} \end{array}$$

where the response variable *y* represents the predicted N indicator (LDM, LAI, or LNA), and *x* represents the VI used for N-status estimation (NDRE, RERVI, NDVI, or RVI). The parameters *a*_0_, *a*_1_, *b*_0_, *b*_1_, *b*_2_, *c*_0_, *c*_1_, *d*_0_, and *d*_1_ were estimated using the least square method implemented in the R language environment (R Core Team).

The coefficient of determination (*R*^2^) for each model was calculated to assess the calibration performance. *R*^2^ provides a measure of how well-observed outcomes are replicated by the model, based on the proportion of total variation of outcomes explained by the model (Draper and Smith, 2014). However, *R*^2^ is not suitable to evaluate the predictive and fitting performance among models with different forms and different numbers of parameters, due to the risk of overfitting for models with higher *R*^2^ but more parameters.

Therefore, the Akaike Information Criterion (AIC) were further used for model selection in this study. The basic viewpoint of AIC is that the selected model is intended to accurately predict future data rather than to infer the “true distribution” of the calibration data (Akaike, 1974; Shmueli, 2010). Since the true predictive performance of a fitted model depends largely on the number of free parameters of the model, the AIC can be used to select model by penalizing for a large number of parameters and discouraging overfitting (Akaike, 1974; Bozdogan, 1987). The AIC is calculated using Equation 5.

(5) $$\begin{array}{r} \text{AIC}=\;2k-2\text{ln}\left(\hat{L}\right) \end{array}$$

where *k* is the number of estimated parameters in the model. In this study, including the terms of residual, *k* was 3 for linear, exponential, and power regressions and 4 for quadratic regression. $\hat{L}$ is the maximum likelihood of the model for the data.

AICs of all the single VI-based regression models were calculated using function AIC in R language environment, which were further verified using self-complied function. The lowest value of AIC indicates the preferable model. The scatter diagrams of the selected VI-based models were plotted using GraphPad Prism 6 (GraphPad Software Inc., San Diego, CA, USA). With *R*^2^ and AIC, stepwise multiple linear regression models based on spectral reflectance of R, Re, and NIR bands were also evaluated to estimate LDM, LAI, and LNA.

Prediction for N indicators was further conducted in the independent validation datasets using the models above. The practical validation performance of the models was estimated by comparing *R*^2^, relative root mean square error (RRMSE, %) and relative error (RE, %) between the predicted variable and the true observed variable. The higher the *R*^2^ and the lower the RRMSE and RE, the higher the precision and accuracy of the model for predicting plant N indicators. The formulas of RRMSE and RE are listed as below:

(6) $$\begin{array}{r} \text{RRMSE }\left(\%\right)=\frac{100}{\bar{O_{i}}}\;\times \;\sqrt{\frac{1}{n}\;\times \;\overset{n}{\underset{i=1}{\sum }}{\left(P_{i}-O_{i}\right)}^{2}} \end{array}$$

(7) $$\begin{array}{r} \text{RE }\left(\%\right)=100\;\times \;\sqrt{\frac{1}{n}\;\times \;\overset{n}{\underset{i=1}{\sum }}(\frac{P_{i}-O_{i}}{O_{i}}{)}^{2}} \end{array}$$

where *P*_*i*_ and *O*_*i*_ are the predicted and observed value of the N indicator (LDM, LAI or LNA), respectively. $\bar{O_{i}}$ is the mean of observed value of the N indicator. *n* is the number of samples.

## Results

### Variability of Rice Leaf N-Status Indicators

Nitrogen-status indicators (LDM, LAI, and LNA) of rice varied greatly across different N rates, management practices, varieties, growth stages, sites, and years (Table 3). For the calibration dataset, the LNA exhibited the most significant variation, with a CV of 73.92%, followed by LAI and LDM with a CV of 60.84 and 59.63%, respectively. Similar results were observed for the calibration dataset and validation datasets of handheld and UAV-based sensing. In total, 624 samples which have a wide range of LDM (51.93 kg ha^−1^ to 5313.40 kg ha^−1^), LAI (0.13 to 10.34), and LNA (0.96 kg ha^−1^ to 192.61 kg ha^−1^) were involved in the calibration experiments. The large variability of these parameters was supposed to cover the major possible situation and make it conceivable to evaluate the potential of using the RapidSCAN sensor for estimating and diagnosing rice leaf N status.

**Table 3:** Descriptive statistics of leaf dry matter (LDM), leaf area index (LAI), and leaf nitrogen accumulation (LNA) across different growth stages, varieties, sites, and years.

**Parameters**	**N**	**Min**	**Max**	**Mean**	**SD**	**CV (%)**
**CALIBRATION DATASET**						
LDM (kg ha^−1^)	624	51.93	5313.40	1991.17	1187.40	59.63
LAI	624	0.13	10.34	3.60	2.19	60.84
LNA (kg ha^−1^)	624	0.96	192.61	58.57	43.29	73.92
**VALIDATION DATASET (HANDHELD)**						
LDM (kg ha^−1^)	168	165.19	3589.63	1580.72	778.92	49.28
LAI	168	0.29	6.84	2.70	1.38	51.23
LNA (kg ha^−1^)	168	5.19	117.38	46.36	24.38	52.60
**VALIDATION DATASET (UAV-BASED)**						
LDM (kg ha^−1^)	192	1238.73	5313.40	2906.11	895.18	30.80
LAI	192	2.25	10.34	5.54	1.74	31.40
LNA (kg ha^−1^)	192	41.75	187.36	97.81	35.50	36.29

### Relationship Between N-Status Indicators and VIs Derived From Handheld System

The relationships between each N-status indicator and each VI were built using handheld data acquired from Experiment 1 and Experiment 2 (Table 4). The performance of using individual VIs to estimate rice LDM, LAI, and LNA varied with the form of selected VI (NDRE, RERVI, NDVI, or RVI) and model type (linear, exponential, power, or quadratic regression model) across different growth stages, treatments, sites, and years.

**Table 4:** *R*^2^ and AIC of the regression models between single VI (NDRE, RERVI, NDVI, or RVI) calculated from handheld sensing data (Experiment 1 and Experiment 2) and each rice N-status indicators (LDM, LAI, or LNA) across different stages of rice growth.

**N Indicator**	**Model**	***R***^****2****^				**AIC**			
		**NDRE**	**RERVI**	**NDVI**	**RVI**	**NDRE**	**RERVI**	**NDVI**	**RVI**
LDM	L	0.73	0.76	0.62	0.72	9797.46	9714.13	10015.26	**9814.69**
	Q	0.77	0.77	0.71	0.72	9700.58	**9689.73**	9837.87	9816.58
	E	0.77	0.76	0.72	0.67	**9690.36**	9724.91	**9813.97**	9912.96
	P	0.76	0.77	0.71	0.72	9711.62	9693.34	9837.56	9818.67
LAI	L	0.73	0.79	0.57	0.69	1938.76	1829.15	2223.73	2024.99
	Q	0.79	0.79	0.68	0.69	1790.44	**1773.23**	2062.38	**2023.95**
	E	0.79	0.77	0.69	0.66	**1783.84**	1774.63	**2053.46**	2061.91
	P	0.78	0.79	0.67	0.69	1872.64	1774.85	2123.53	2025.00
LNA	L	0.74	0.79	0.54	0.69	5646.06	5494.90	6000.31	5755.80
	Q	0.82	0.83	0.67	0.70	5395.70	5368.26	5785.49	5735.20
	E	0.83	0.82	0.70	0.66	**5365.18**	5400.18	**5732.86**	5797.87
	P	0.82	0.83	0.69	0.70	5400.34	**5366.70**	5748.31	**5731.78**

*L, Q, E, and P represent linear, quadratic, exponential, and power regression models, respectively. The numbers in bold represent the best model form for each VI to predict each N indicator based on AIC*.

Based on the AIC values of the models in Table 4, the VIs calculated from NIR and Re reflectance (NDRE and RERVI) had preferable performance across different types of models as compared to VIs calculated from NIR and R reflectance (NDVI and RVI) for estimating LDM, LAI, and LNA, respectively. By comparing the models of the same type (linear, quadratic, exponential, and power models respectively), NDRE and RERVI had higher *R*^2^ values over NDVI and RVI for estimating rice leaf N indicators.

**Figure 3:**
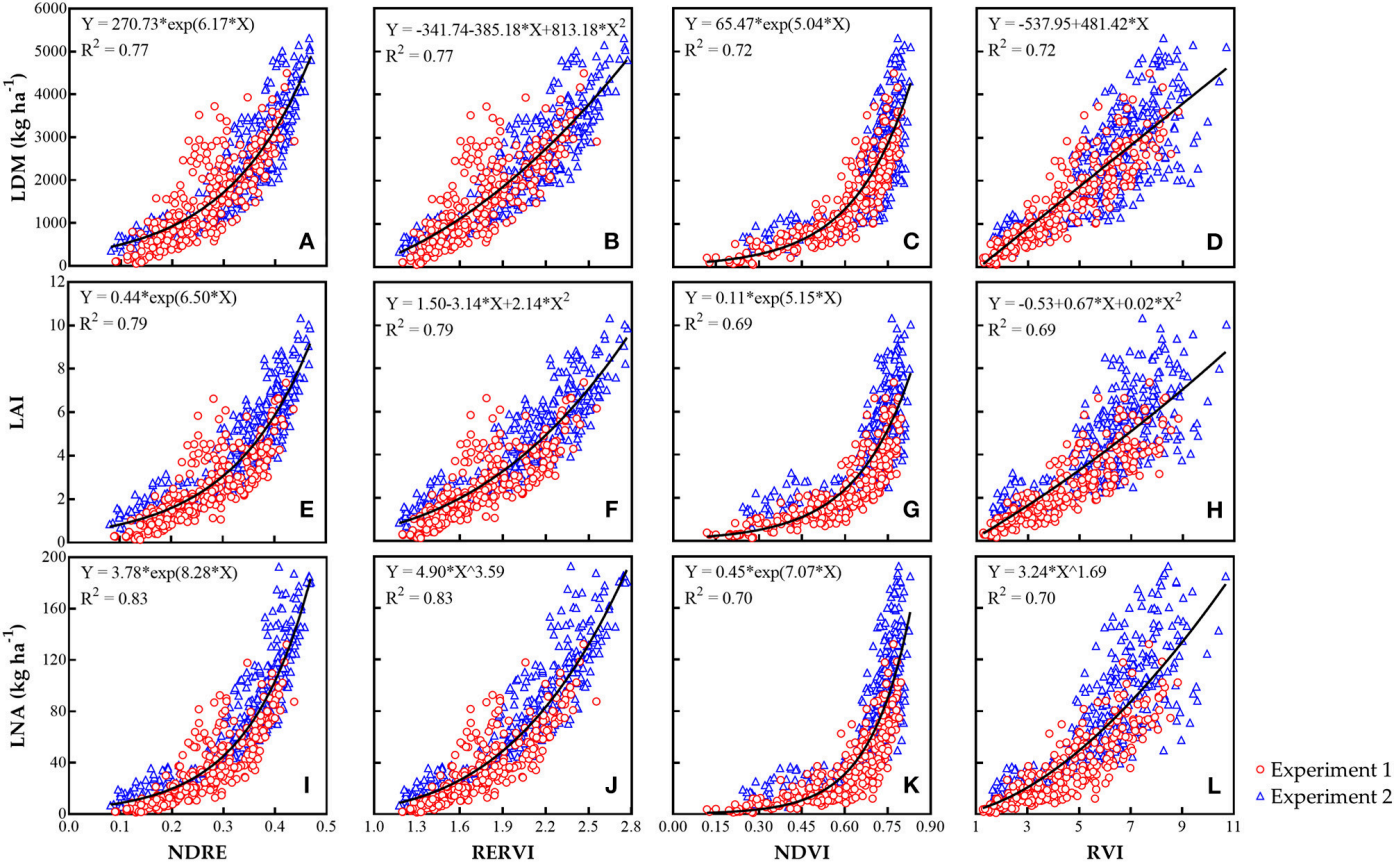
The best relationships based on AIC between each rice leaf N indicator [LDM **(A–D)**, LAI **(E–H)**, and LNA **(I–L)**] and each VI by active canopy sensor RapidSCAN CS-45 across different growth stages, sites, and N treatments from the calibration experiments.

A total of 12 best models were selected for each N indicator and each VI based on AIC. Scatter diagrams of the selected models are presented in Figure 3. Overall, RERVI had great potential for estimating LDM (*R*^2^ = 0.77) and LAI (*R*^2^ = 0.79) with quadratic regression models, and the NDRE had the lowest AIC in exponential model for LNA prediction (*R*^2^ = 0.83). On the other side, obvious saturation effect and relatively poor predictive results were shown by NDVI in the scatter diagrams. For practical application, the predictive models were further evaluated in the following validation analysis.

### Validation of the Relationships Between N-Status Indicators and VIs Using Handheld Data

**Figure 4:**
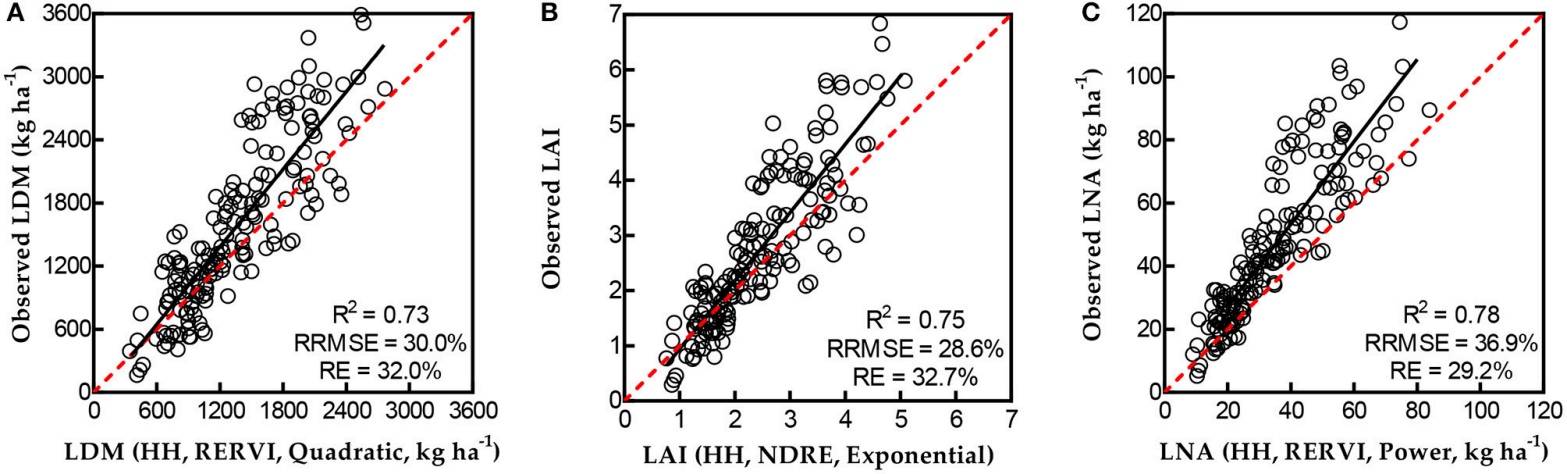
Validation results of LDM **(A)**, LAI **(B)**, and LNA **(C)** for single VI-based predictions using validation datasets of handheld (HH) sensing.

The regression models between VIs and N-status indicators selected in this study were further evaluated with other handheld sensing data acquired from validation experiments (Table 5). The scatter diagrams for the best handheld validation results of VI-based predictions (determined by the lowest RE) are shown in Figure 4.

**Table 5:** Validation results of the selected single VI-based models for estimating N-status indicators with handheld sensing data.

**VI**	**LDM**				**LAI**				**LNA**			
	**Type**	***R*^**2**^**	**RRMSE**	**RE**	**Type**	**R^**2**^**	**RRMSE**	**RE**	**Type**	**R^**2**^**	**RRMSE**	**RE**
NDRE	E	0.73	30.5%	36.0%	E	0.75	28.6%	32.7%	E	0.78	37.2%	29.8%
RERVI	Q	0.73	30.0%	32.0%	Q	0.75	29.2%	33.1%	P	0.78	36.9%	29.2%
NDVI	E	0.47	42.4%	55.2%	E	0.41	50.0%	64.6%	E	0.48	45.7%	55.3%
RVI	L	0.46	41.8%	56.2%	Q	0.40	48.4%	65.0%	P	0.47	42.8%	51.7%

*L, Q, E, and P represent linear, quadratic, exponential, and power regression models, respectively*.

In the validation results between the predicted and observed N indicators, comparing NDRE with NDVI and RERVI with RVI, the VIs calculated from NIR and Re reflectance had lower RRMSE and RE than the VIs calculated from NIR and R reflectance. This showed the great potential of NDRE and RERVI for N-status estimation.

Considering the relatively slight variation of RRMSE across different VI-based models to estimate each N-status indicator, REs were compared to evaluate the models for the best handheld data-based validation (Figure 4). For LDM, RERVI performed the best using a quadratic regression model (RRMSE = 30.0%, and RE = 32.0%) in validation. While for LAI estimation, using an exponential regression model, NDRE performed better than other selected models (RRMSE = 28.6%, and RE = 32.7%). RERVI had the best validation performance by using a power regression model to estimate LNA (RRMSE = 36.9%, and RE = 29.2%).

### Validation of the Models Between N-Status Indicators and VIs Using UAV-Based Data

**Figure 5:**
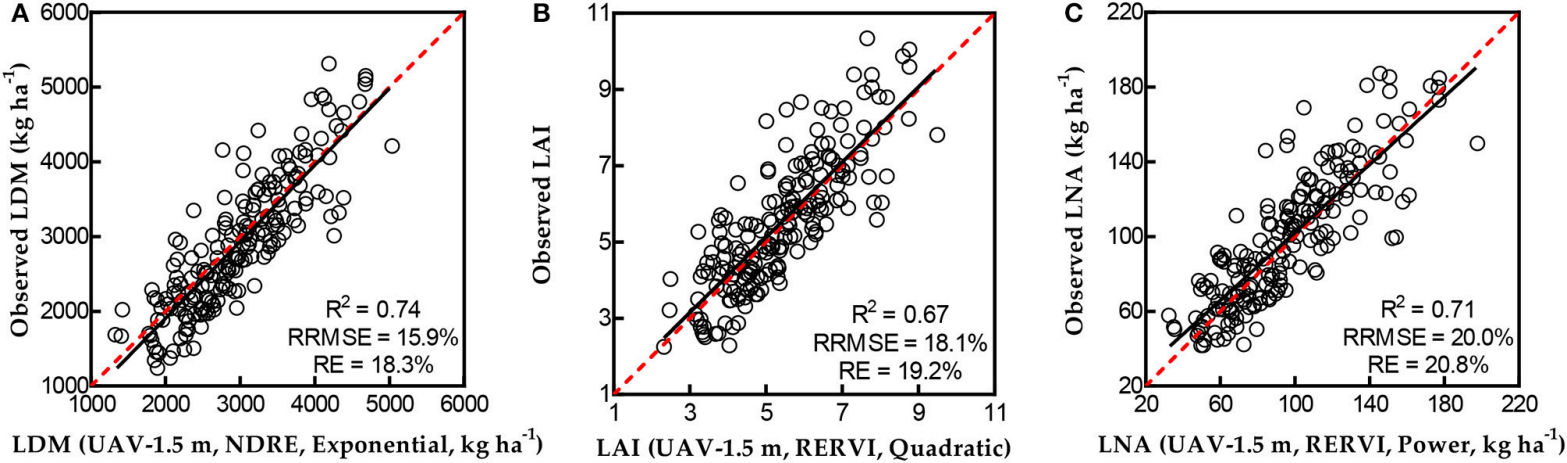
Validation results of LDM **(A)**, LAI **(B)**, and LNA **(C)** for single VI-based predictions using validation datasets of UAV-based sensing.

All the selected models were also evaluated with UAV-based data from validation experiments for testing the feasibility of applying the UAV-based RapidSCAN CS-45 for monitoring rice leaf N-status with models derived from handheld data (Table 6). The UAV-based data contain two parts which were achieved from the UAV-based sensing with a 1.5 and 2 m height above the rice canopy, respectively. Scatter diagrams for UAV-based validation results of VI-based predictions with the best models (determined by the lowest RE) are shown in Figure 5.

**Table 6:** Validation results of the selected single VI-based models for estimating rice N-status indicators with UAV-based sensing data.

**VI**	**LDM**				**LAI**				**LNA**			
	**Type**	**R^**2**^**	**RRMSE**	**RE**	**Type**	**R^**2**^**	**RRMSE**	**RE**	**Type**	**R^**2**^**	**RRMSE**	**RE**
**VALIDATION DATA: UAV-1.5 M**												
NDRE	E	0.74	15.9%	18.3%	E	0.67	18.0%	19.7%	E	0.71	19.8%	21.0%
RERVI	Q	0.74	16.0%	18.6%	Q	0.67	18.1%	19.2%	P	0.71	20.0%	20.8%
NDVI	E	0.49	23.0%	24.9%	E	0.46	24.5%	25.6%	E	0.48	29.5%	31.6%
RVI	L	0.51	23.9%	25.1%	Q	0.48	25.8%	26.4%	P	0.49	31.7%	32.5%
**VALIDATION DATA: UAV-2 M**												
NDRE	E	0.45	24.7%	28.8%	E	0.35	26.7%	32.5%	E	0.38	30.6%	37.0%
RERVI	L	0.45	23.8%	28.7%	Q	0.35	26.3%	31.5%	P	0.38	31.0%	36.9%
NDVI	E	0.31	26.4%	26.6%	E	0.25	29.1%	30.1%	E	0.30	33.0%	31.7%
RVI	L	0.31	26.7%	26.6%	Q	0.25	29.6%	30.4%	P	0.30	33.9%	31.4%

*L, Q, E, and P represent linear, quadratic, exponential, and power regression models, respectively*.

Compared with the validation performance of models from 2 m UAV-based data, VIs calculated from the same bands of 1.5 m UAV-based data better estimated LDM, LAI, and LNA. Hence, the best validation results were all from 1.5 m UAV-based datasets. This showed that the height of 1.5 m above the rice canopy is a suitable flight height for monitoring rice leaf N-status with the UAV-based RapidSCAN CS-45 sensor.

Similar to the validation results of handheld data, the results of *R*^2^, RRMSE, and RE reveal that NDRE and RERVI had better performance than NDVI and RVI from the 1.5 m height sensing dataset. Determined by the REs, the best validation result for LDM estimation was presented in Figure 5 with NDRE-based exponential model (RRMSE = 15.9%, and RE = 18.3%). While RERVI performed the best for LAI (RRMSE = 18.1%, and RE = 19.2%) and LNA (RRMSE = 20.0%, and RE = 20.8%) monitoring in the UAV-based validation result based on the quadratic model and power model, respectively.

### Stepwise Multiple Linear Regression Analysis

The handheld sensing data from the calibration experiments were pooled together to build stepwise regression models with reflectance of NIR, R, and Re bands to estimate N-status indicators in the calibration experiments (Table 7). The results indicated that 74.6% of LDM variability could be explained with NIR and Re bands. The first chosen band for LAI was also the NIR band, followed by the Re band (*R*^2^ = 0.751). In contrast to the results of LDM and LAI, the R band was also chosen in the stepwise multiple linear regression models to estimate LNA after NIR and Re bands, and 77.6% of the variability of LNA could be explained. As one of the input selection rules of stepwise multiple linear regression, the AIC of the models decreased following the steps for LDM, LAI, and LNA estimation. The calibration and validation results (Table 8) based on AIC indicated that these models did not perform better than the best VI-based models, as shown above.

**Table 7:** Stepwise multiple linear regression models based on RapidSCAN CS-45 bands (R, Re, and NIR, %) for estimating rice N-status indicators across growth stages.

**N Indicator**	**Step**	**Regression equation**	**R^**2**^**	**AIC**
LDM	1	199.902 * NIR-5466.137	0.734	9784.10
(kg ha^−1^)	2	154.231 * NIR-182.546 * Re-136.377	0.746	9757.17
LAI	1	0.370 * NIR-10.189	0.739	1915.78
	2	0.285 * NIR-0.336 * Re-0.379	0.751	1888.42
LNA	1	7.393 * NIR-217.223	0.755	5599.62
(kg ha^−1^)	2	5.834 * NIR-6.229 * Re - 35.348	0.766	5574.12
	3	6.932 * NIR-6.767-Re + 2.259 * R-84.355	0.776	5549.28

*R, Re, and NIR represent reflectance (%) of red (670 nm), red edge (730 nm), and near-infrared (780 nm) bands, respectively*.

**Table 8:** Validation results of the stepwise multiple linear regression models based on spectral reflectance of RapidSCAN CS-45 wavebands (R, Re, and NIR) for estimating rice N-status indicators with data acquired from handheld sensing, and UAV-based sensing of a 1.5 or 2 m height above the rice canopy.

**N-status indicators**	**R^**2**^**	**RRMSE**	**RE**
**VALIDATION DATA: HANDHELD (EXPERIMENT 3 AND EXPERIMENT 4)**			
LDM (kg ha^−1^)	0.73	27.6%	32.2%
LAI	0.74	26.9%	32.4%
LNA (kg ha^−1^)	0.80	38.9%	58.2%
**VALIDATION DATA: UAV-1.5 M (EXPERIMENT 2)**			
LDM (kg ha^−1^)	0.71	18.1%	20.7%
LAI	0.65	20.8%	21.3%
LNA (kg ha^−1^)	0.69	22.5%	23.1%
**VALIDATION DATA: UAV-2 M (EXPERIMENT 2)**			
LDM (kg ha^−1^)	0.38	24.9%	29.8%
LAI	0.29	27.0%	32.3%
LNA (kg ha^−1^)	0.27	31.4%	39.5%

## Discussion

### Potential of UAV-Based Active Sensing for Rice N-Status Monitoring

UAV-based active sensing, as a new exploration of the low-altitude sensing method, is expected to be outstanding for crop N-status monitoring. But for testing the feasibility of the sensing system, it is critical to consider its superiorities compared to traditional methods and its actual performance from experimental results.

Ground-based sensing using handheld or ground vehicle-mounted spectrometers is recognized to be capable of describing crop trait expression and nutrient monitoring (Saberioon et al., 2014; Yang et al., 2017); however, the data collection instability, low sensing efficiency, and high cost are its limiting factors (Yang et al., 2017). The paddy field condition has an influence on the sensing speed stability of ground-based sensing, which leads to spectral data variability and instability, and the footprints or wheel ruts may increase the influence and cause roots injured. The low sensing efficiency was shown in an example where more than 40 h were needed to collect 20,000 plots' spectral data on single rows using a single vehicle at speed about 0.56 m per second, and it would be more time-consuming by handheld sensing (about 0.5 m per second walking in paddy fields; White et al., 2012). By contrast, UAV-based sensing has advantages regarding the sensing stability (controlled with an automatic ground station program), high sensing efficiency (e.g., with a 2 m per second heading aerial speed in this study), and relatively low cost (Yang et al., 2017).

Traditional UAV-based sensing focuses on image-based utilization, and relevant studies differ a lot over the sensor use and crop species. UAV image-based sensing for crop monitoring is related to several issues like image capturing and mosaicing, geometric correction, spectral radiation processing, and useful feature extraction (Zhang and Kovacs, 2012; Yang et al., 2017). These complex processes have critical effects on the data compatibility, are time-consuming, and are closely related to the operator's experience (Zhang and Kovacs, 2012). The standard utilization process and data output form are still lacking for the practical utilization of UAV image-based sensing for crop nitrogen estimation, which limits the eurytopic model building and high throughout plant-phenotypic data analysis using diverse datasets from different studies. By collecting standard spectral reflectance directly without data processing, UAV-based active sensing has convenience regarding data collection, and it is easy-to-use for common consumers compared to image-based sensing. In addition, a low sensing height offers the UAV-based active sensing with potential ability to collect more spectral information under canopy surface compared to UAV-based passive monitoring (Holland et al., 2012; Yang et al., 2017).

For testing the actual performance of UAV-based active sensing for rice leaf N monitoring, RapidSCAN CS-45 was chosen in this study. According to the manufacturer's instructions, the sensor can distinguish its own light signal from that of surrounding ambient light by modulating the light source (by rapidly pulsing the light source on and off many times a second). Therefore, this technology named PSR measurement ensures a degree of data stability within an effective sensing distance (0.3–3 m above the canopy as introduced by the manufacturer), and this leads to the possibility of building universal predictive models for handheld and UAV-based sensing.

From the study of Krienke et al. (2017), a highly significant linear relationship was found between NDRE from handheld and UAV-based RapidSCAN sensing on a maize canopy, yet, the handheld data showed less variation as compared to UAV-based data collected with a fluctuating height from 0.5 to 1.5 m. Therefore, in this study, AIC-based optimum predictive models were built based on handheld datasets to estimate rice LDM (*R*^2^ = 0.77), LAI (*R*^2^ = 0.79), and LNA (*R*^2^ = 0.83), and it was also validated by independent handheld and UAV-based data for testing the model performance and the transferability of handheld-based sensing models to the UAV-based active sensing. The results showed great potential both for handheld and UAV-based sensing.

### Evaluation of N-Status Prediction and Analysis for Saturation Effect of NDVI

An obvious saturation effect was observed with NDVI for crop N-status prediction as shown in Figure 3. NDVI saturation is a common view in previous studies. For example, NDVI achieved by the active canopy sensor GreenSeeker (Trimble Navigation Limited, Sunnyvale, CA, USA) could explain 80% of wheat biomass variability (Cao et al., 2015); however, the saturation effect of NDVI is obviously existent. The applicability of NDVI for crop nutrition monitoring is probably due to the high transmittance of the NIR band and a degree of function of the red band (Knipling, 1970). This leads to the saturation effect of NDVI (Thenkabail et al., 2000).

Therefore, using wavelengths with similar penetration into the plant canopy may be one of the methods employed to overcome the NDVI saturation problems (Niel and McVicar, 2004). The existing research has shown that the saturation problem could be reduced by the Re band, and Re-based VIs could be better correlated with crop N status (Delegido et al., 2013). Re-radiation penetrates deeper into crop canopies due to lower absorption by chlorophyll compared to radiation at the red waveband. Therefore, the sensitivity of Re reflectance is higher than R reflectance (Kanke et al., 2012; Zhou et al., 2018). Considering the four kinds of vegetation indices selected in this study for rice N-status monitoring, the VIs calculated from NIR, and Re reflectance (NDRE or RERVI) performed better than the VIs calculated from NIR and R reflectance (NDVI or RVI) both in handheld and UAV-based datasets. With similar results, Re-based vegetation indices using Crop Circle ACS-470 sensor and satellite remote sensing images improved plant concentration and uptake estimation for maize (Li et al., 2014). Moreover, NDRE and RERVI also performed more stably in UAV-based experiments in this study.

Another method consists in using ratio vegetation indices. The saturation effect is related to the normalization effect from the formula of normalized VIs, which could be avoided by the ratio VI to some degree (Gnyp et al., 2014). According to Cao et al. (2015), RVI significantly reduced the saturation effect of NDVI for estimating aboveground biomass of wheat. Based on linear models (Table 4), when comparing RVI with NDVI and RERVI with NDRE, the ratio VIs performed better for the N indicator prediction as compared to the normalized VIs (calculated by the same wavebands) in this study.

The RVI based on NIR and Re band (RERVI) was a great choice which combined two sides above. According to the potato experiment conducted by Zhou et al. (2018), a linear relationship was found between N concentration and RERVI, and this agrees with the fact that RERVI increased linearly with canopy chlorophyll content, as indicated by radiative transfer models from Clevers and Kooistra (2012). RERVI also had a good performance in calibration results compared to other VIs with linear regression models in this study.

For considering the three wavebands together, stepwise multiple linear regression analysis (SMLR) was conducted for N-status monitoring. Nevertheless, the multiple regression models did not perform significantly better than the best VI-based models. As shown by the stepwise linear regression results from the research by Cao et al. (2015), with Crop Circle ACS-470 employed on wheat across all growth stages, 53% of aboveground biomass variability and 67% of plant N uptake could be explained with two to three bands. However, the validation results also indicated that the stepwise linear regression models did not perform better than the best VI-based models. This is probably for the reason that only two or three bands were used for SMLR. If four to ten discrete wavebands could be used for SMLR, the performance of N-status monitoring would be significantly improved (Nguyen et al., 2007; Cao et al., 2013).

Besides, not only SMLR, but also some other models or methods such as support vector machines (SVM), artificial neural networks (ANN), and random forest (RF), have also been proved to have potential for monitoring crop N-status(Noh et al., 2006; Wang et al., 2013; Liang et al., 2015). Superior methods (e.g., SVM, ANN, or RF) need to be tested in future studies of combining various spectral, spatial, and environmental information for a better crop N-status prediction.

### Considerations for Practical Utilization of UAV-Based Active Sensing

This study has shown that the UAV mounted active canopy sensor is feasible for monitoring rice N-status, yet some points, including sensing distance, canopy perturbance from air movement, and the slightly unstable flight condition caused by the aerodynamic ground effect of a low-altitude flight, still need to be addressed for the practical use of this sensing system.

The first issue is the suitable sensing distance which differs a lot over sensor types. For instance, spectral data of Crop Circle ACS-210 were befittingly collected on an aircraft at an altitude of 3–5 m above ground level on corn (Lamb et al., 2009). The suitable measuring height was between 0.4 and 1.2 m above the wheat canopy with a passive sensor mounted on a sensor support on a UAV (Ni et al., 2017). The sensing distance is directly reflected in the flight height above the canopy in this study, as the sensor posture has been fixed by the gimbal. Our validation results of the UAV-based dataset, which indicates that the height of 1.5 m above the rice canopy is much more suitable for rice N-status monitoring than 2 m, were in consensus with the result of distance sensitivity study on the turf grass canopy with UAV-mounted RapidSCAN CS-45 by Krienke et al. (2017). And this shows that spectral reflectance was affected by sensing distance and the UAV-based RapidSCAN sensor operated effectively within a range of 0.5–1.5 m above the canopy.

**Figure 6:**
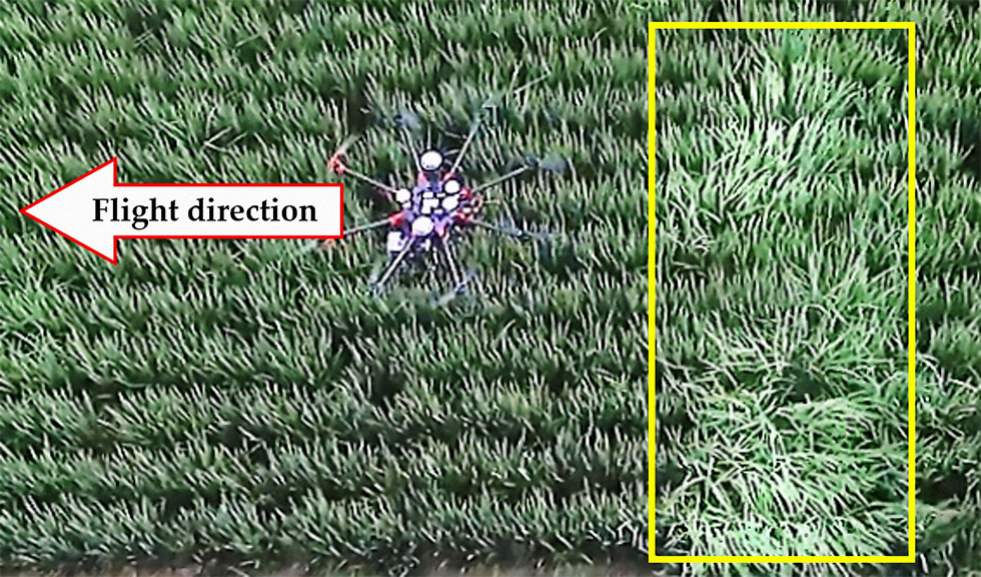
Top view and practical sensing state of the UAV-based sensing system with RapidSCAN CS-45. The height of the sensor under the UAV is 1.5 m above the rice canopy and flight speed in the heading direction is 2 m/s. The arrow symbol shows the heading flight direction of the UAV. The yellow box with increased brightness shows the perturbed canopy area generated by the UAV via a visual check.

Canopy perturbance from air movement generated by the UAV is also crucial to be considered while using UAV based data acquisition. The factors influencing canopy perturbance are numerous, which include but are not limited to flight height, speed, posture, surrounding air condition and air velocities influenced by aircraft design. In the study of a UAV-based passive sensor by Ni et al. (2017), after numerical computational fluid dynamics simulations, solar sensors, and two-band sensors were designed and fixed on the two ends of a long sensor support to avoid the down-wash flow field below the UAV and the system should be applied in a hovering state. However, long sensor support is not suitable for this study considering the dynamic flight state and the sensor character. In view of the technical restriction, computational fluid dynamics simulations were not conducted in this study. While, as shown in Figure 6, via several attempts by visual checks, the perturbed canopy area (marked by the yellow box) was at the back of the sensed area when the UAV was controlled to aviate with a height of above 1.5 m and heading speed of over 2 m/s.

Besides, sometimes a slightly unstable flight condition would be caused by the aerodynamic ground effect of a low-altitude flight, even though the effect is much lighter on multi-rotor UAVs than single-rotor helicopters (Sanchez-Cuevas et al., 2017). Real-Time Kinematic GPS (RTK GPS) is a powerful technology, which can provide centimeter-level high accuracy 3D positioning of UAVs (Spockeli, 2015). Therefore, to overcome the unstable condition caused by the aerodynamic ground effect, an RTK system designed for UAV is highly recommended. As shown in the UAV commissioning before the actual experiments in this study, flight condition is much more stable with the DJI D-RTK GNSS System than non-RTK utilization.

The height of 1 m above the rice canopy was also considered in the first test of UAV-based active sensing in Experiment 2. However, obvious canopy perturbance was generated under the UAV in that sensing mode. Moreover, the flight condition was unstable with a 1 m-height setting, and the flight height fluctuated from about a 0.5 to 1.2 m height above the canopy even with the D-RTK GNSS system. For data stability and experiment security, UAV-based sensing at a 1 m-height was canceled in the following tests. This unstable flight condition was probably caused by mixed reasons of the aerodynamic ground effect and the technical lack of accurate aerial positioning for proximal flight.

## Conclusion

The calibration and validation results showed the great potential of active canopy sensor RapidSCAN CS-45 to monitor rice leaf N-status using both handheld and UAV-mounted modes. Great transferability of handheld-based predictive models to UAV-based sensing was verified by the UAV data-based validation experiment. Based on model evaluation and selection by AIC, 77, 79, and 83% of the variability in LDM, LAI, and LNA were explained with the optimal VI-based regression models derived from the calibration datasets, respectively. Considering different data acquired from UAV-based sensing and handheld sensing, NDRE and RERVI exhibited a much better performance in estimating rice N-status than the traditional R-based vegetation indices (NDVI and RVI), which also displayed great potential in overcoming the saturation problem of NDVI.

The present study has put forward a novel way of monitoring rice leaf N-status by the application of a multi-rotor unmanned aerial vehicle with a portable active canopy sensor. The height of 1.5 m above the rice canopy with a heading speed of 2 m/s was suitable for practical use. Future investigations are still needed to consider the combined effect of flight height, speed, canopy perturbance, ground effect, and new low-altitude location technology. Additionally, the entire automation workflow of data collection, processing for N status prediction, and management need to be developed for this sensing system in the future.

## Author Contributions

QC, YT, YZ, and WC conceived and designed the experiments. SL, XD and QK performed the experiments. SL and QC analyzed the data and wrote the original manuscript. SA, TC, XL, YT, YZ, and WC reviewed and revised the manuscript. All authors read and approved the final manuscript.

### Conflict of Interest Statement

The authors declare that the research was conducted in the absence of any commercial or financial relationships that could be construed as a potential conflict of interest.
